# A cohort study of the effects of social support on cerebral cardiovascular disease in subjects with metabolic syndrome

**DOI:** 10.1371/journal.pone.0305637

**Published:** 2024-07-18

**Authors:** Sung-Kyung Kim, Yong Whi Jeong, Dae Ryong Kang, Jang Young Kim, Hunju Lee, Sang-baek Koh

**Affiliations:** 1 Department of Preventive Medicine, Wonju College of Medicine, Yonsei University, Wonju, Korea; 2 Department of Medical Informatics and Biostatistics, Graduate School, Yonsei University, Wonju, Korea; 3 Department of Precision Medicine, Wonju College of Medicine, Yonsei University, Wonju, Korea; 4 Department of Internal Medicine, Yonsei University Wonju College of Medicine, Wonju, Korea; Kurume University School of Medicine, JAPAN

## Abstract

**Introduction:**

Previous studies have extensively examined the relationship between social support and various health outcomes. However, little is known about the distinct longitudinal associations between perceived social support and the development of cardiovascular events in patients with metabolic syndrome. In this cohort study, we investigated whether the levels of perceived social support in patients with metabolic syndrome were associated with an increased risk of cerebrovascular and cardiovascular events.

**Methods:**

The level of social support was assessed using the Medical Outcomes Study-Social Support Survey (MOS-SSS) in 2,721 individuals living in Wonju and Pyeongchang, South Korea. The presence of metabolic syndrome was determined by physical measurements and blood tests, and the occurrence of cerebral cardiovascular disease in relation to the presence of metabolic syndrome and the level of social support was analyzed using Cox proportional-hazards models.

**Results:**

The median follow-up period was 2,345 days (2,192–2,618). Overall, in the group with metabolic syndrome and low social support, low social support was associated with an increased risk of later cerebral cardiovascular events; in this group, the hazard ratio after adjusting for confounding variables was 1.97 times (95% confidence interval, 1.01–3.85) higher than that in the group without metabolic syndrome and low social support.

**Conclusion:**

This study shows, for the first time, that the level of social support is a risk factor for preventing cerebral cardiovascular disease in patients with metabolic syndrome and suggests that social support status should be incorporated into multifactorial risk assessment and intervention procedures to prevent metabolic syndrome and cerebral cardiovascular disease.

## Introduction

According to a 2019 study, ischemic heart disease and stroke were the leading causes of disability-adjusted life years in both 50 to 74 and over 75 age groups [1]. Metabolic syndrome (MetS) is known to be associated with an increased risk of myocardial infarction and stroke. Traditional cardiovascular risk formulas are inadequate for determining cardiovascular risk in younger people and women [2], and MetS, which constitutes a set of factors representing the highest risk level for heart attack, is a useful alternative for assessing cardiovascular risk [3]. Despite various efforts to reduce the prevalence of MetS, the global prevalence of the disease is estimated to be approximately one-fourth of the world’s population, equivalent to more than one billion people, and the prevalence of MetS is increasing worldwide. South Korea is no exception, with the most recent statistics showing that the prevalence of MetS increased from 24.5% in 2008 to 28.1% in 2017 in men and from 18.7% in 2008 to 20.5% in 2017 in women [4]. A 2021 study in South Korea found that among the five components of MetS, the prevalence of hypertension and high fasting blood glucose levels has been increasing since 2007 in both men and women [5]. This increase in the incidence of MetS has been shown to increase deaths from cardiovascular disease and is predicted to increase the societal burden of morbidity from these diseases [6–9]. Thus, mitigating the cerebrovascular risk factors associated with MetS may be one approach to reducing the individual and population risk of cardiovascular events.

Since MetS itself is a cluster of risk factors for cerebrovascular disease, efforts to reduce the adverse effects of its complications may require not only lifestyle changes, such as exercise and diet [10, 11], but also the identification of other causes that may influence the development and course of the syndrome. Many risk factors associated with MetS have been identified, but its prevalence continues to increase. For example, in a recent study, we suggested that pesticide exposure may play a role in the development of MetS in rural populations [12]. Recent studies have shown that lower socioeconomic status is associated with a more unfavorable metabolic health profile [13], and various studies have suggested that the health effects of low socioeconomic status may depend on the presence of social support [14–16]. Previous studies have reported that lower levels of social support are associated with higher mortality rates due to chronic diseases [17]. However, no study has analyzed the risk of developing cerebrovascular disease in relation to the level of social support in patients with MetS. Therefore, using the Medical Outcomes Study-Social Support Survey (MOS-SSS), which was developed to determine the level of social support in patients with common chronic diseases [18], this study aimed to determine whether the development of cerebral cardiovascular disease differed in relation to the level of social support in patients with MetS.

## Methods

We used data from the Korean Genome and Epidemiology Study on Atherosclerosis Risk of Rural Areas in the Korean General Population (KoGES-ARIRANG), a population-based prospective cohort study, to assess the prevalence, incidence, and risk factors of chronic degenerative disorders such as MetS, hypertension, diabetes, and cardiovascular disease. KoGES-ARIRANG invited all adults aged 40–70 years residing in the rural areas of Wonju and Pyeongchang, South Korea, to participate in the study. Demographic shifts are infrequent in this area, and the population can be followed up in the long term [19, 20]. The cohort study began on December 6, 2005, and written informed consent was obtained from participants before the study began. The baseline study ended on December 31, 2008. Subsequently, the follow up study began on January 1, 2009 and ended on December 31, 2011. We accessed this cohort data on September 20, 2017. To obtain cerebral cardiovascular disease information, which was the endpoint of this study, medical record information of those who participated in the KoGES-ARIANG study was provided by the Korea Disease Control and Prevention Agency in connection with the data of the Korea National Health Insurance Service.

**Fig 1:**
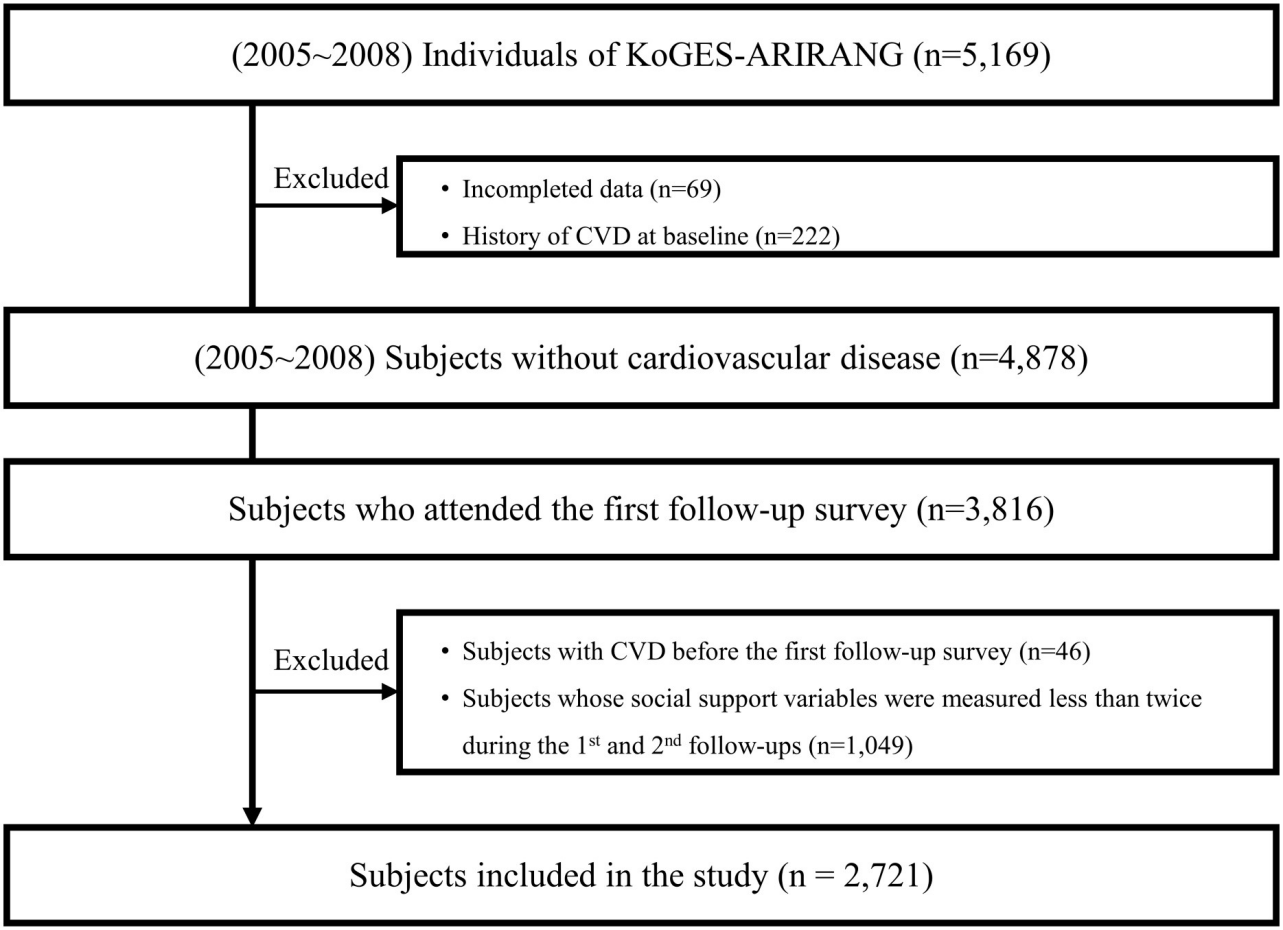
Flow chart of study design.

The baseline survey, conducted between November 2005 and February 2008, included 5,169 adults aged 40–70 years. We excluded 69 participants with incomplete data and 222 participants with a history of cerebral cardiovascular disease at baseline. The number of participants who underwent the first follow-up was 3,816. Forty-six individuals who had cerebral cardiovascular disease before the first follow-up and 1,049 individuals who underwent measurement of social support variables less than twice during the first and second follow-ups were excluded. The total sample consisted of 2,721 participants (Fig 1). The study was approved by the Institutional Review Board (IRB) of Wonju Severance Christian Hospital and the requirement for informed consent was obtained (IRB No. CR105024).

### Data collection

At baseline and follow-up, the study participants completed a standardized medical history and lifestyle questionnaire and underwent a comprehensive health examination according to standard procedures. Body weight and height were measured while the participants wore light indoor clothing without shoes. Body mass index (BMI) was calculated as the ratio of an individual’s weight to height squared (kg/m^2^). Waist circumference was measured in the horizontal plane midway between the inferior margin of the ribs and the superior border of the iliac crest using a tape measure (SECA-200; SECA, Hamburg, Germany). Current smoking was defined as when participants had smoked ≥100 cigarettes in their lifetime and had reported “currently smoking” in the questionnaire. Participants who answered yes to the question, “Do you usually drink alcohol?” were defined as alcohol consumers. Participants who answered no to the question, “Do you perform physical exercise regularly enough to make you sweat?” were assigned to the non-exercise group. The questionnaire also evaluated the participants’ current marital status. Systolic and diastolic blood pressures were measured twice on the right arm using a standard mercury sphygmomanometer (Baumanometer; Copiague, NY, USA). With the participants seated, an appropriately sized cuff was applied snugly around the upper right arm at the heart level. The cuff size for each participant was chosen according to the mid-arm circumference. Two measurements were taken with at least 5-min intervals in between, and the mean of the two measurements was used in the analyses. According to the Eighth Joint National Committee (JNC-8) guidelines [21], hypertension was defined as systolic blood pressure of at least 140 mmHg, diastolic blood pressure of at least 90 mmHg, or current use of antihypertensive agents. Diabetes mellitus was defined as a fasting serum glucose level of at least 126 mg/dL or the current use of blood glucose-lowering agents at baseline. Serum concentrations of total cholesterol, high-density lipoprotein (HDL) cholesterol, low-density lipoprotein (LDL) cholesterol, and triglycerides were determined using enzymatic methods (Advia 1650; Siemens, Tarrytown, NY) [19, 20, 22].

### Definition of MetS and the Medical Outcomes Study-Social Support Survey

MetS was defined in accordance with the harmonized definition for MetS [23] as the presence of at least three of the following criteria: (1) abdominal obesity, defined as waist circumference ≥90 cm for men or ≥85 cm for women (following Korean-specific cutoffs for abdominal obesity defined by the Korean Society of Obesity) [24]; (2) hypertriglyceridemia, defined as serum triglyceride concentration ≥150 mg/dL (1.69 mmol/L); (3) low HDL cholesterol, defined as serum HDL cholesterol concentration <40 mg/dL (1.04 mmol/L) for men or <50 mg/dL (1.29 mmol/L) for women; (4) high blood pressure, defined as systolic blood pressure ≥130 mmHg, diastolic blood pressure ≥85 mmHg, or treatment with antihypertensive agents; and (5) high fasting glucose, defined as fasting serum glucose level ≥100 mg/dL or previously diagnosed type 2 diabetes.

The Medical Outcomes Study-Social Support Survey (MOS-SSS) is a self-reported questionnaire consisting of 20 items rated on a 5-point Likert-type scale from 1 (never) to 5 (always) [18]. The first item reports the size of the social network, and the subsequent 19 items measure four dimensions of functional social support: emotional support (expression of positive affect, empathetic understanding, and encouragement of expressions of feelings); informational support (provision of advice, information, guidance, or feedback); tangible support (provision of material or behavioral assistance); affectionate support (including expressions of love and affection); and positive social interaction (the availability of other people to do fun things with you). This score was converted to a possible range of 0–100, with higher scores indicating greater support. This social support score, with higher scores indicating more support, was converted to a possible range of 0–100, and the Spanish version was used for score conversion [25].

To determine the effects of MetS and the changes in social support on the occurrence of cerebral cardiovascular disease, social support scores measured in the first and second follow-up surveys were used. The MOS-SSS score measured in the first and second follow-ups was classified into four quartiles, and participants who were consecutively in the fourth quartile in the first and second follow-ups were defined as a consistently low group, while the rest of the participants were defined as a group with no low level of social support. Participants were classified into four groups according to changes in social support and MetS: (1) without MetS and without persistently low social support; 2) without MetS and with persistently low social support; 3) with MetS and without persistently low social support; and 4) with MetS and with persistently low social support.

### Endpoint definition

The study endpoint was cerebral cardiovascular disease, defined as myocardial infarction, angina, or stroke within the follow-up period. A stroke was defined as a case in which CT or MRI was performed under ICD-10 codes I60-I69 or G45-46. Myocardial infarction was identified as a case in which the ICD-10 code was I21-I25, lab codes were B2640, C3941, C3942, CY277, CY278, and CY279, or codes for procedures such as surgery, percutaneous intervention, and angiography were included. Angina was defined as a case in which coronary recanalization was performed using ICD-10 code I20.

### Statistical analysis

Categorical variables were described as numbers and percentages, and continuous variables were summarized as means and standard deviations. Differences among groups were analyzed using analysis of variance (ANOVA) with Scheffé’s post-hoc analysis method for continuous variables and chi-square tests for categorical variables. Cumulative cerebral cardiovascular disease incidence rates were estimated using the Kaplan–Meier survival curve. Log-rank tests were conducted to determine the differences in the cumulative incidence of cerebral cardiovascular disease among the groups. To investigate the association between cerebral cardiovascular disease incidence, the presence of MetS, and changes in social support, Cox proportional-hazard analysis was performed to estimate the hazard ratios (HRs) and 95% confidence intervals (CIs). Three models with progressive degrees of adjustment were used. First, the analysis was performed based on the presence of MetS and changes in social support. Second, we adjusted for age and sex. Finally, we further adjusted for LDL cholesterol level, smoking, and regular exercise. Statistical significance was set at p <0.05. All statistical analyses were conducted using SAS (version 9.4; Cary, NC, USA) and R 4.3.1 (Institute for Statistics and Mathematics, Vienna, Austria).

## Results

### Characteristics of the participants

Clinical characteristics of the study participants in relation to the presence of MetS and changes in social support are shown in Table 1. The total number of participants was 2,721. Specifically, among participants without MetS, 1,614 were classified into the without persistently low social support group, and 163 were classified into the persistently low social support group. Furthermore, among participants with MetS, 841 were categorized into the without persistently low social support group and 103 were categorized into the with persistently low social support group. Compared to the other three groups, those with MetS and with persistently low social support were older; had higher BMI and total cholesterol, LDL cholesterol, and triglyceride levels; showed a greater incidence of hypertension and diabetes; and were more likely to not exercise regularly.

**Table 1:** Baseline characteristics of the study population.

Variables	Without MetS and without persistently low social support	Without MetS and with persistently low social support	With MetS and without persistently low social support	With MetS and with persistently low social support	p-value
**Participants, n**	1,614	163	841	103	
**Age (years)**	54.43 (8.04)^b^^,^^c^	54.71 (8.58)	56.41 (7.75)^d^	57.06 (7.58)^d^	<0.001
**Sex (men)**	988 (61.21)	100 (61.35)	460 (54.70)	68 (66.02)	0.007
**BMI (kg/m** ^**2**^ **)**	23.69 (2.69)^b^^,^^c^	23.67 (2.80)^b^^,^^c^	26.48 (2.95)^a^^,^^d^	26.53 (3.33)^a^^,^^d^	<0.001
**Waist circumference (cm)**	80.44 (7.82)^b^^,^^c^	80.73 (8.11)^b^^,^^c^	89.36 (7.28)^a^^,^^d^	89.09 (7.27)^a^^,^^d^	<0.001
**Current smoking**	256 (15.86)	25 (15.34)	115 (13.67)	15 (14.56)	0.553
**Alcohol intake**	652 (40.40)	72 (44.17)	367 (43.64)	34 (33.01)	0.116
**No regular exercise**	1,096 (67.97)	116 (71.17)	540 (64.21)	91 (88.35)	<0.001
**Systolic BP (mmHg)**	126.02 (16.60)^b^^,^^c^	126.64 (17.58)^b^^,^^c^	139.96 (17.12)^a^^,^^d^	138.49 (19.30)^a^^,^^d^	<0.001
**Diastolic BP (mmHg)**	80.03 (10.84)^b^^,^^c^	79.90 (10.97)^b^^,^^c^	88.08 (10.45)^a^^,^^d^	89.10 (12.96)^a^^,^^d^	<0.001
**Hypertension**	179 (11.09)	23 (14.11)	330 (39.24)	48 (46.60)	<0.001
**Diabetes mellitus**	64 (3.97)	6 (3.68)	130 (15.46)	18 (17.48)	<0.001
**Total cholesterol (mg/dL)**	199.67 (37.96)^b^^,^^c^	192.65 (32.20)^b^^,^^c^	206.53 (38.53)^a^^,^^d^	212.81 (40.43)^a^^,^^d^	<0.001
**HDL cholesterol (mg/dL)**	48.89 (11.01)^b^^,^^c^	47.19 (9.31)^b^^,^^c^	40.63 (8.46)^a^^,^^d^	41.69 (8.80)^a^^,^^d^	<0.001
**LDL cholesterol (mg/dL)**	117.58 (32.13)	113.68 (27.73)	120.60 (34.10)	123.43 (34.42)	0.014
**Triglyceride (mg/dL)**	115.68 (65.50)^b^^,^^c^	107.67 (59.97)^b^^,^^c^	212.34 (147.19)^a^^,^^d^	216.76 (143.45)^a^^,^^d^	<0.001

Data are presented by n (%) or mean (SD). MetS, metabolic syndrome; BP, blood pressure; BMI, body mass index; HDL, high-density lipoprotein; low-density lipoprotein.

^a^ p-value <0.05 vs. without MetS and with persistently low social support after analysis of variance (ANOVA) followed by Scheffé’s post-hoc comparison;

^b^ p-value <0.05 vs. with MetS and without persistently low social support after ANOVA followed by Scheffé’s post-hoc comparison;

^c^ p-value <0.05 vs. with MetS and with persistently low social support (+) after ANOVA followed by Scheffé’s post-hoc comparison;

^d^ p-value <0.05 vs. without MetS and persistently low social support after ANOVA followed by Scheffé’s post-hoc comparison.

#### Kaplan–Meier analysis of time to disease in relation to MetS and social support

**Fig 2:**
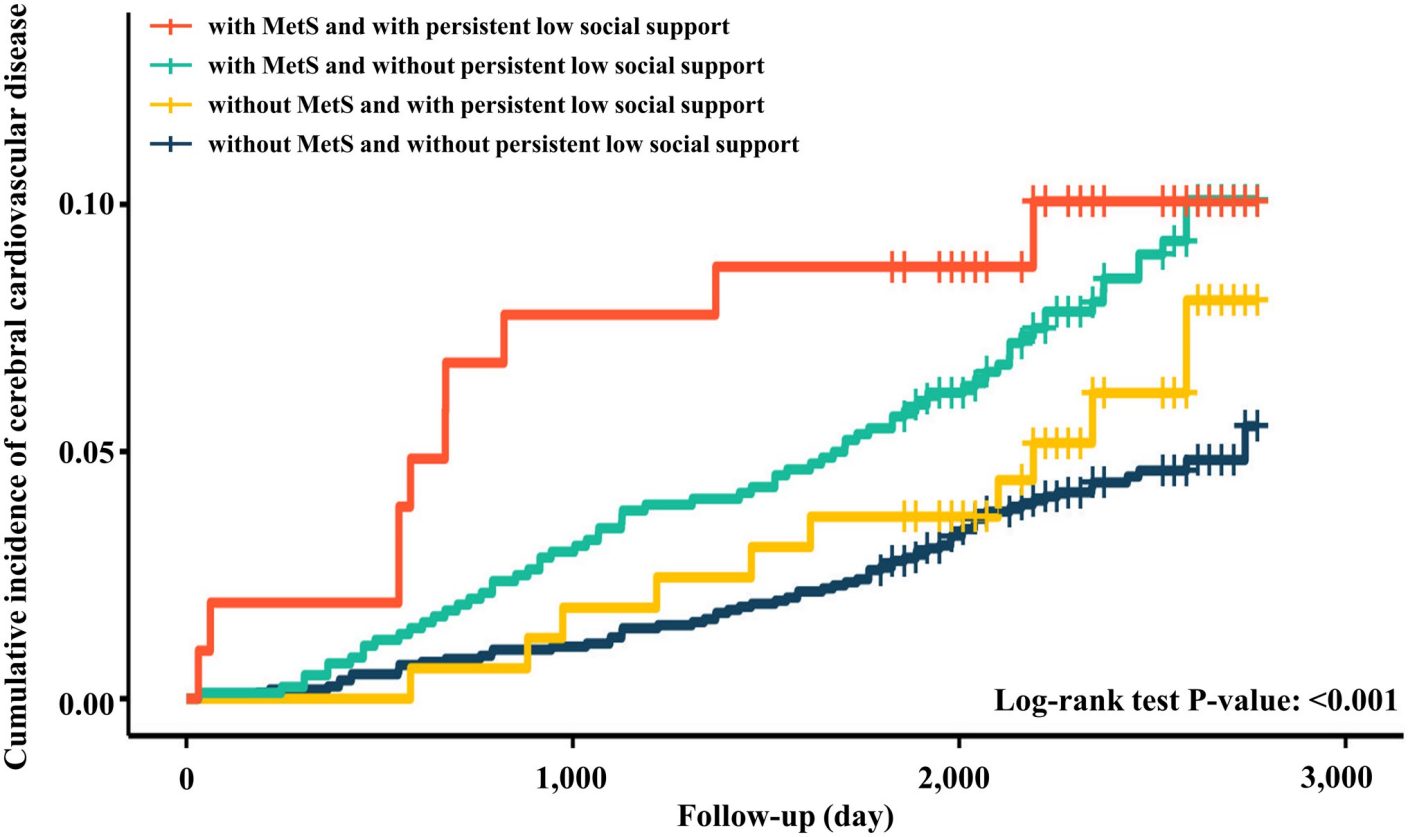
Kaplan-Meier analysis of cerebral cardiovascular disease according to the MetS and social support.

Of the 2,721 participants analyzed in our study, 162 (5.95%) developed cerebral cardiovascular disease during the follow-up period. The Kaplan–Meier survival curves for cumulative incidence from cerebral cardiovascular disease in relation to changes in social support and the presence of MetS are shown in Fig 2. In comparison with the other three groups, those with MetS and those with persistently low social support showed a higher probability of developing incident cerebral cardiovascular disease. The cumulative incidence of cerebral cardiovascular disease showed significantly higher in the group without MetS and persistently low social support (log-rank test, p <0.05).

### Relationship between cerebral cardiovascular disease events, the presence of MetS, and changes in social support

Cox proportional-hazard analysis was used to investigate the risk of incident cerebral cardiovascular disease in relation to the presence of MetS and changes in social support (Table 2). The incidence of cerebral cardiovascular disease was the highest at 16.10 (95% CI, 7.72–29.61) in the patients with MetS and with persistently low social support. The crude HR for cerebral cardiovascular disease was 2.41 (95% CI, 1.24–4.67) in the group with MetS and with persistently low social support in comparison to the group without MetS and persistently low social support. In this group, the HR for cerebrovascular events was 1.97 (95% CI, 1.01–3.85) after adjustment for age, sex, low-density lipoprotein cholesterol level, smoking, and lack of regular exercise. In addition, even in the group with MetS and without persistently low social support, the crude HR was significant at 1.98 (95% CI, 1.43–2.76). The fully adjusted HR was 1.78 (95% CI, 1.28–2.48). Additionally, Cox proportional-hazards analysis was used to examine the risks of myocardial infarction (MI), angina, and stroke, and similar trends were observed when stratified by cerebral cardiovascular disease (Table 3). The HRs for incident myocardial infarction, angina, and stroke in the groups with and without persistently low social support were 2.06 (95% CI, 1.05–4.00), 2.14 (95% CI, 1.10–4.16), and 1.94 (95% CI, 0.99–3.77), respectively, which were higher than the HRs in the groups with and without persistently low social support.

**Table 2:** Hazard ratios for incident cerebral cardiovascular disease.

	Without MetS and without persistently low social support	Without MetS and with persistently low social support	With MetS and without persistently low social support	With MetS and with persistently low social support
**Total (N = 2,721)**	1,614	163	841	103
**Number of incidence cases (%)**	71 (4.40%)	10 (6.13%)	71 (8.44%)	10 (9.71%)
**Person-years**	10,491.35	1,048.49	5,326.23	621.02
**Incident rate (Per 1,000 person-year)**	6.77 (5.29–8.54)	9.54 (4.58–17.55)	13.33 (10.41–16.82)	16.10 (7.72–29.61)
**Crude HR**	1.00 (Ref)	1.41 (0.73–2.74)	**1.98 (1.43–2.76)**	**2.41 (1.24–4.67)**
**Model 1**	1.00 (Ref)	1.36 (0.70–2.64)	**1.76 (1.27–2.45)**	**2.08 (1.07–4.04)**
**Model 2**	1.00 (Ref)	1.35 (0.70–2.62)	**1.78 (1.28–2.48)**	**1.97 (1.01–3.85)**

HR, hazard ratio; MetS, metabolic syndrome; Ref, reference; CI, confidence interval. Model 1: adjusted for age and sex. Model 2: adjusted for age, sex, low-density lipoprotein cholesterol level, smoking, and lack of regular exercise.

**Table 3:** Hazard ratios for incident MI, angina, and stroke according to the presence of metabolic syndrome and changes in social support.

	Without MetS and without persistently low social support	Without MetS and with persistently low social support	With MetS and without persistently low social support	With MetS and with persistently low social support
**MI**				
Number of incidence cases (%)	34 (2.11%)	6 (3.68%)	33 (3.92%)	5 (4.85%)
Person-years	10,582.97	1,056.49	5,424.04	640.44
Incident rate (per 1,000 person-year)	3.21 (2.23–4.49)	5.68 (2.09–12.37)	6.08 (4.19–8.54)	7.81 (2.54–18.23)
Crude HR	1.00 (Ref)	1.51 (0.78–2.93)	**2.03 (1.46–2.82)**	**2.41 (1.24–4.67)**
Model 1	1.00 (Ref)	1.48 (0.76–2.88)	**1.80 (1.29–2.50)**	**2.13 (1.01–4.13)**
Model 2	1.00 (Ref)	1.47 (0.76–2.86)	**1.81 (1.30–2.52)**	**2.06 (1.05–4.00)**
**Angina**				
Number of incidence cases (%)	7 (0.43%)	1 (0.61%)	11 (1.31%)	4 (3.88%)
Person-years	10,660.94	1,067.49	5,491.16	652.95
Incident rate (per 1,000 person-year)	0.66 (0.26–1.35)	0.94 (0.24–5.22)	2.00 (1.00–3.58)	6.13 (1.67–15.71)
Crude HR	1.00 (Ref)	1.54 (0.79–2.99)	**2.04 (1.47–2.84)**	**2.41 (1.24–4.67)**
Model 1	1.00 (Ref)	1.53 (0.79–2.97)	**1.82 (1.31–2.53)**	**2.19 (1.13–4.26)**
Model 2	1.00 (Ref)	1.52 (0.78–2.96)	**1.83 (1.31–2.54)**	**2.14 (1.10–4.16)**
**Stroke**				
Number of incidence cases (%)	41 (2.54%)	4 (2.45%)	43 (5.11%)	3 (2.91%)
Person-years	10,571.24	1,064.25	5,420.53	654.70
Incident rate (per 1,000 person-year)	3.88 (2.78–5.26)	3.76 (1.02–9.63)	7.93 (5.74–10.69)	4.58 (0.95–13.41)
Crude HR	1.00 (Ref)	1.46 (0.75–2.83)	**2.01 (1.45–2.79)**	**2.34 (1.21–4.53)**
Model 1	1.00 (Ref)	1.44 (0.74–2.79)	**1.80 (1.29–2.50)**	**2.01 (1.04–3.91)**
Model 2	1.00 (Ref)	1.43 (0.74–2.77)	**1.81 (1.30–2.52)**	1.94 (0.99–3.77)

HR, hazard ratio; MetS, metabolic syndrome; Ref, reference; CI, confidence interval. Model 1: adjusted for age and sex. Model 2: adjusted for age, sex, low-density lipoprotein cholesterol level, smoking, and lack of regular exercise.

## Discussion

To the best of our knowledge, this is the first study to examine the influence of social support on the development of cerebral cardiovascular disease in subjects with MetS. MetS is a contributing factor to cerebral cardiovascular disease, as described above, and needs to be actively managed. In this study, participants were evaluated based on their metabolic syndrome status and quartile scores of social support. Since we confirmed that there was no interaction between social support score and metabolic syndrome, we were able to evaluate dividing into groups (S1 Table). After adjusting for confounding variables, the hazard ratio for cerebral cardiovascular events in the group with MetS and with persistently low social support was 1.97 times (95% CI 1.01–3.85) higher than in the group without MetS and persistently low social support. In the group without MetS and with persistently low social support, also showed a 1.35-fold higher HR compared to the reference group, but this was not statistically significant (95% CI 0.70–2.62) (Table 2). In this respect, our study suggests that high levels of social support may reduce the morbidity associated with cerebral cardiovascular events in subjects with MetS. In other words, with the global increase in the incidence of MetS, a thorough understanding of the factors that predict the development of cerebral cardiovascular disease events in these subjects, especially modifiable factors such as social support, has become increasingly important.

Social support is a key element of patients’ self-care skills [26]. A study led by Williams and Bond found a correlation between social support and patient self-management activities and showed that social support has a positive impact on patients’ psychosocial comfort [27]. If patients better understand the correlation between their illness and psychological factors and have access to social support, they can more easily improve their attitudes toward their illness (e.g., from pessimistic to optimistic and from passive to active). Consequently, disease states can improve through social support [28]. Therefore, combining medical management with social support has become a growing trend [28]. However, guidelines related to public social support, interpersonal affective support, and positive social interactions specific to individuals with MetS are limited.

The Chronic Care Model (CCM) is a comprehensive system model with medical and social benefits for patients and healthcare providers [29–31]. Using this model as a reference, some local governments, such as Seoul, South Korea, are running social support programs for MetS, which include education, medical expense support, and free counseling [32].

Implementing social support models, including CCM, in the community requires multidisciplinary teamwork in an organized and collaborative manner. To work optimally, policymakers need to consider a range of factors related to system design, including differences in socioeconomic factors, access to healthcare, and available technology [31]. This may not be easily achievable in countries where primary care is still developing because of a lack of resources [33] and budgetary issues related to social support. In addition, the MOS-SSS includes items related to affective support and positive social interactions, which cannot be improved by individual efforts alone. Therefore, improvement of these items should be promoted through the organization of health clubs and linkages with care facilities related to MetS in the community, the development of guidelines, patient education, and social campaigns [34–36].

The effect of social support on cardiovascular disease in patients with MetS can be inferred from previous studies. First, patients with high levels of affectional support and positive social interactions were more likely to experience better physical activity [37]. General social support appears to support psychological adjustment to chronic diseases such as coronary heart disease (CHD), and perceived social support at the individual level has an independently significant effect on health-related quality of life in patients with CHD [38, 39]. We found that the low social support group was older, had higher BMI, total cholesterol, LDL cholesterol, and triglyceride levels, higher rates of hypertension and diabetes, and was more likely not to exercise regularly. These are mostly known risk factors for cerebral cardiovascular disease, and our findings are consistent with previous studies (Table 1) [40, 41]. Lower levels of perceived social support have also been associated with higher mortality, particularly in cardiovascular disease [17]. Our findings extend the results of these studies and propose that the level of social support in patients with MetS may modify their risk of developing cerebrovascular diseases.

This study had a few limitations. First, participants were community-dwelling adults aged 40 years and older; therefore, these associations need to be replicated in a more diverse population to generalize our findings to clinical populations. Second, because the MOS-SSS is self-reported, there are concerns that participants may over- or under-report their perceived level of social support; however, the MOS-SSS has previously established reliability and validity [42]. Third, owing to the relatively short follow-up period, we may not have fully captured the development of cerebrovascular diseases in our study population. Fourth, subjects with consistently low social support were defined as those who repeatedly fell into the 1^st^ quartile in the first and second surveys. Since there was a difference in the interval between the first and second survey periods measuring social support, and the distribution of the social support was left-skewed, subjects consistently belonging to 1^st^ quartile were defined as the group with low social support (S2 Table). Logistic regression and Cox proportional-hazard analysis were performed on the impact of social support by quartile on the development of cerebral cardiovascular disease. Although not statistically significant, we confirmed a tendency for social support to be positively associated with the incidence of cerebral cardiovascular disease only in the 1st quartile, regardless of the survey time point (S3 Table). Since we focused only on subjects with low social support due to limitations in data measurement, it is necessary to use social support as a continuous variable in future studies to determine whether there is any association with the occurrence of cerebral cardiovascular diseases in various distributions. Finally, it is possible that the increased HR in the group with persistently low social support was secondary to risk factors associated with cerebral cardiovascular disease. As described previously, we found that the persistently low social support group was older, had higher BMI, total cholesterol, LDL cholesterol, and triglyceride levels, had higher rates of hypertension and diabetes, and was more likely not to exercise regularly. To minimize these effects, we performed statistical analyses that adjusted for age, LDL cholesterol levels and lack of regular exercise, excluding BMI, triglyceride levels, hypertension, and diabetes, which are associated with main components of the MetS.

In conclusion, the current study confirmed that among subjects with MetS, low levels of social support are associated with an increased risk of developing cerebral cardiovascular disease. To the best of our knowledge, this is the first report of this type. The findings of our study emphasize the driving force behind creating a supportive and beneficial social environment in patients with MetS; an important aspect in this context is that social support is a modifiable factor associated with cerebral cardiovascular disease prevention. Thus, our findings can serve as a basis for potential plans to reverse the increasing prevalence of cerebral cardiovascular disease and lower the burden of cerebral cardiovascular disease in Korea.

## Supporting information

S1 Table Hazard ratios for incidence of cerebral cardiovascular disease by social support level and presence of MetS. (DOCX)

S2 Table Baseline characteristics of the study population by social support level. (DOCX)

S3 Table Odds ratios and Hazard ratios for the presence of MetS and incidence of cerebral cardiovascular disease by social support level. (DOCX)
