# Supplementary material for: A cohort study of the effects of social support on cerebral cardiovascular disease in subjects with metabolic syndrome
Source: PLoS One. 2024 Jul 18;19(7):e0305637. doi: 10.1371/journal.pone.0305637 (PMC11257245; doi:10.1371/journal.pone.0305637)
Supplement: S1 Table — (DOCX) [file pone.0305637.s001.docx]

| S table 1. Hazard ratios for incidence of cerebral cardiovascular disease by social support level and presence of MetS | | |
| --- | --- | --- |
|  | HR (95% CI) | P-value |
| First survey |  |  |
| MetS | 0.69 (0.21-2.22) | 0.531 |
| Social support | 0.99 (0.98-1.00) | 0.084 |
| MetS*Social support | 1.01 (0.99-1.03) | 0.070 |
| Second survey |  |  |
| MetS | 2.84 (0.84-9.59) | 0.093 |
| Social support | 1.00 (0.99-1.01) | 0.766 |
| MetS*Social support | 1.00 (0.98-1.01) | 0.533 |
| Average of first and second surveys |  |  |
| MetS | 1.12 (0.30-4.77) | 0.808 |
| Social support | 1.00 (0.98-1.01) | 0.387 |
| MetS*Social support | 1.01 (0.99-1.02) | 0.473 |
| HR, hazard ratio; CI, confidence interval. | | |
