# Supplementary material for: A cohort study of the effects of social support on cerebral cardiovascular disease in subjects with metabolic syndrome
Source: PLoS One. 2024 Jul 18;19(7):e0305637. doi: 10.1371/journal.pone.0305637 (PMC11257245; doi:10.1371/journal.pone.0305637)
Supplement: S2 Table — (DOCX) [file pone.0305637.s002.docx]

| S table 2. Baseline characteristics of the study population by social support level | | | | | | | | |  |
| --- | --- | --- | --- | --- | --- | --- | --- | --- | --- |
|  |  | Prevalence of MetS | |  |  | Incidence of cerebral cardiovascular disease | | | |
|  | Total | no | yes | P-value |  | Total | no | yes | P-value |
| Social support in First survey |  |  |  | 0.732 |  |  |  |  | 0.355 |
| q4 (97.37-100.00) | 753 (27.67) | 482 (28.71) | 271 (28.71) |  |  | 753 (27.67) | 704 (27.51) | 49 (30.25) |  |
| q3 (84.21-97.37) | 620 (22.79) | 415 (23.35) | 205 (21.72) |  |  | 620 (22.79) | 588 (22.98) | 32 (19.75) |  |
| q2 (65.79-84.21) | 638 (23.45) | 417 (23.47) | 221 (23.41) |  |  | 638 (23.45) | 606 (23.68) | 32 (19.75) |  |
| q1 (0.00-65.79) | 710 (26.09) | 463 (26.06) | 247 (26.17) |  |  | 710 (26.09) | 661 (25.83) | 49 (30.25) |  |
| Social support in Second survey |  |  |  | 0.974 |  |  |  |  | 0.871 |
| q4 (95.39-100.00) | 997 (36.64) | 348 (36.86) | 649 (36.52) |  |  | 997 (36.64) | 934 (36.50) | 63 (38.89) |  |
| q3 (84.87-95.39) | 307 (11.28) | 108 (11.44) | 199 (11.20) |  |  | 307 (11.28) | 289 (11.29) | 18 (11.11) |  |
| q2 (70.39-84.87) | 744 (27.34) | 253 (26.80) | 491 (27.63) |  |  | 744 (27.34) | 704 (27.51) | 40 (24.69) |  |
| q1 (0.00-70.39) | 673 (24.73) | 235 (24.89) | 438 (24.65) |  |  | 673 (24.73) | 632 (24.70) | 41 (25.31) |  |
| Persistently social support |  |  |  | 0.533 |  |  |  |  | 0.568 |
| q4 (97.37-100.00) | 385 (37.56) | 244 (37.03) | 141 (38.52) |  |  | 385 (37.56) | 361 (37.60) | 24 (36.92) |  |
| q3 (88.16-97.37) | 85 (8.29) | 56 (8.50) | 29 (7.92) |  |  | 85 (8.29) | 81 (8.44) | 4 (6.15) |  |
| q2 (67.76-88.16) | 217 (21.17) | 148 (22.46) | 69 (18.85) |  |  | 217 (21.17) | 206 (21.46) | 11 (16.92) |  |
| q1 (0.00-67.76) | 338 (32.98) | 211 (32.02) | 127 (34.70) |  |  | 338 (32.98) | 312 (32.50) | 26 (40.00) |  |
| Data are presented by n (%) or min-max. Persistently social support had subjects in the same quartile for the first and second surveys. | | | | | | | | | |
