# Supplementary material for: A cohort study of the effects of social support on cerebral cardiovascular disease in subjects with metabolic syndrome
Source: PLoS One. 2024 Jul 18;19(7):e0305637. doi: 10.1371/journal.pone.0305637 (PMC11257245; doi:10.1371/journal.pone.0305637)
Supplement: S3 Table — (DOCX) [file pone.0305637.s003.docx]

| S table 3. Odds ratios and Hazard ratios for the presence of MetS and incidence of cerebral cardiovascular disease by social support level | | | | | |
| --- | --- | --- | --- | --- | --- |
|  | MetS |  | Cerebral cardiovascular disease | | |
|  | OR (95% CI) |  | OR (95%CI) |  | HR (95%CI) |
| Social support in the First survey |  |  |  |  |  |
| q4 | Ref. |  | Ref. |  | Ref. |
| q3 | 0.88 (0.70-1.10) |  | 0.78 (0.49-1.24) |  | 0.76 (0.48-1.18) |
| q2 | 0.94 (0.76-1.18) |  | 0.76 (0.48-1.20) |  | 0.74 (0.48-1.16) |
| q1 | 0.95 (0.77-1.18) |  | 1.07 (0.71-1.61) |  | 1.03 (0.69-1.53) |
| Social support in the Second survey |  |  |  |  |  |
| q4 | Ref. |  | Ref. |  | Ref. |
| q3 | 1.01 (0.77-1.32) |  | 0.92 (0.54-1.59) |  | 0.94 (0.56-1.59) |
| q2 | 0.96 (0.79-1.17) |  | 0.84 (0.56-1.27) |  | 0.88 (0.59-1.30) |
| q1 | 1.00 (0.82-1.23) |  | 0.96 (0.64-1.44) |  | 1.00 (0.67-1.48) |
| Persistently social support |  |  |  |  |  |
| q4 | Ref. |  | Ref. |  | Ref. |
| q3 | 0.90 (0.55-1.47) |  | 0.74 (0.25-2.20) |  | 0.71 (0.25-2.03) |
| q2 | 0.81 (0.57-1.15) |  | 0.80 (0.39-1.67) |  | 0.83 (0.41-1.70) |
| q1 | 1.04 (0.77-1.41) |  | 1.25 (0.71-2.23) |  | 1.25 (0.72-2.17) |
| Persistently social support had subjects in the same quartile for the first and second surveys.  OR, odds ratio; HR, hazard ratios; CI, confidence interval; Ref, reference. | | | | | |
